# Harnessing CRISPR-dCas13Rx to identify novel antisense targets for therapeutic splicing modulation

**DOI:** 10.1016/j.omtn.2026.102895

**Published:** 2026-03-22

**Authors:** Ravindra N. Singh, Christiano R.R. Alves

**Affiliations:** 1 Department of Biomedical Sciences, Iowa State University, Ames, IA 50011, USA; 2 Department of Pathology, University of Pittsburgh, Pittsburgh, PA 15261, USA

## Main text

Pre-mRNA splicing is a fundamental process that removes introns and generates mRNAs in higher eukaryotes. The mechanism of pre-mRNA splicing involves a combinatorial control that varies from intron to intron and is governed by interdependent *cis*-regulatory elements. Canonical sequence motifs at the beginning and the end of introns define the 5′ splice site (5′ss) and 3′ss, respectively. Regulatory *cis*-elements away from the splice sites include exonic splicing enhancers (ESEs), exonic splicing silencers (ESSs), intronic splicing enhancers (ISEs), intronic splicing silencers (ISSs), and structural elements. Any given sequence stretch within a pre-mRNA may harbor multiple overlapping *cis*-elements in a structural context that defines the specificity. While mutations in the canonical splice site motifs account for ∼10% of human genetic diseases,^1^ mutations leading to abrogation of regulatory *cis*-elements away from the splice sites account for an additional 25% of all human genetic diseases.^2^ Currently, there are no simple treatment options for most of these genetic disorders, although therapeutic approaches such as antisense oligonucleotides (ASOs) and small molecules have been emerging as strong candidates to treat some of these conditions.

Spinal muscular atrophy (SMA) provided the first proof of principle that an ASO-based therapy aimed at the prevention of exon skipping could be developed by targeting sequences away from the actual site of the pathogenic mutation.^3^ However, progress toward developing similar therapies for other genetic disorders has been hampered due to the lack of reliable and cost-effective methods to identify targetable splicing regulatory elements. Recent reports demonstrate the usefulness of Cas13 variants paired with specific guide RNAs (gRNAs) in splicing modulation through targeting specific sequences within pre-mRNAs.^4^^,^^5^^,^^6^ This approach takes advantage of a catalytically inactive Cas13 (dCas13) variant that binds to, but does not cleave, the target RNA sequence. One utility of this system is the screening of multiple gRNA sequences overlapping pathogenic mutations for their effect directly on pre-mRNA splicing. This approach could be particularly valuable if the splicing connection of a pathogenic mutation could be paired with the target discovery for an ASO-induced splicing correction. For example, more than eighty missense mutations of *POLR3A* are associated with pathological conditions, including a broad spectrum of neurological disorders.^7^ However, it is not known how many of these mutations cause aberrant splicing and if any of these splicing-associated mutations could be corrected.

**Figure 1:**
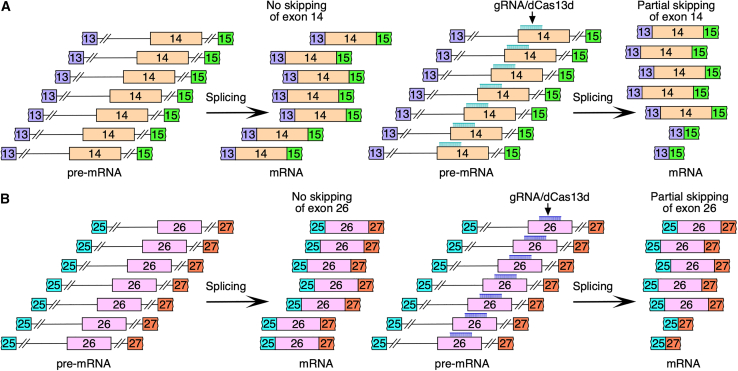
Splicing of exons 14 and 26 of *POLR3A* in the presence of gRNAs complexed with dCas13d Experiments measured splicing of wild-type endogenous transcripts of *POLR3A*. Diagram not to the scale. (A) Left and right images represent splicing of *POLR3A* exon 14 in the absence and presence of gRNAs, respectively. gRNAs targeting exonic positions known to harbor pathogenic mutations trigger modest skipping of exon 14. (B) Left and right images represent splicing of *POLR3A* exon 26 in the absence and presence of gRNAs, respectively. gRNAs targeting exonic positions known to harbor pathogenic mutations trigger modest skipping of exon 26.

**Figure 2:**
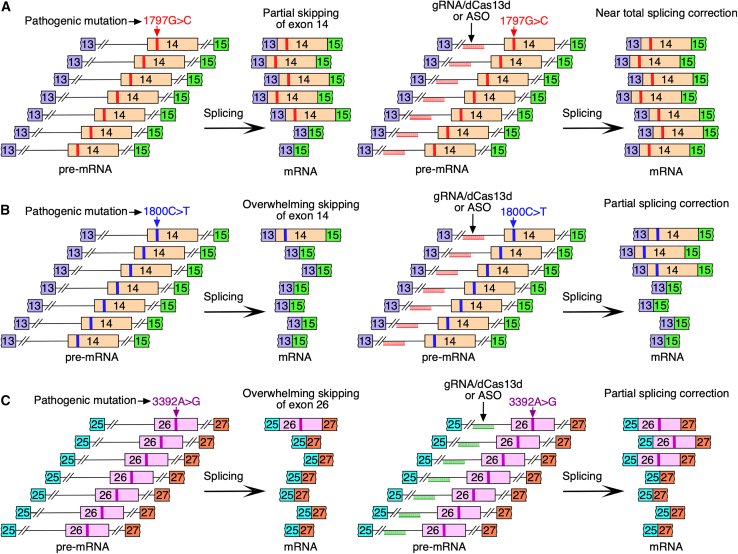
Correction of splicing of pathogenic mutations of exons 14 and 26 of *POLR3A* in the context of minigenes Diagram not to the scale. (A) Left image represents splicing of *POLR3A* exon 14 carrying pathogenic 1797G>C mutation. Right image represents near total splicing correction of exon 14 carrying 1797G>C mutation by gRNA or ASO targeting intronic sequences upstream of exon 14. (B) Left image represents splicing of *POLR3A* exon 14 carrying pathogenic 1800C>T mutation. Right image represents modest splicing correction of exon 14 carrying 1800C>T mutation by gRNA or ASO targeting intronic sequences upstream of exon 14. (C) Left image represents splicing of *POLR3A* exon 26 carrying pathogenic 3392A>G mutation. Right image represent modest splicing correction of exon 26 carrying 3392A>G mutation by gRNA or ASO targeting intronic sequences upstream of exon 26.

In this issue of *Molecular Therapy Nucleic Acids*, Shkreta et al. demonstrated the utility of the dCas13/gRNA system to determine the link between missense mutations of *POLR3A* and aberrant splicing.^8^ They performed an initial screening in EcR-293 cells transfecting a dCas13rx variant paired with ten different gRNAs to target*POLR3A* sequences and overlap pathogenic missense mutations across eight exons.^8^ Two of the gRNAs, one targeting exon 14 and the other targeting exon 26, triggered their skipping, supporting that the exonic sequences being targeted harbor ESEs (Figure 1). By extension, results also suggested that ∼20% of the pathogenic missense mutations of *POLR3A* abrogate ESEs and/or create ESSs. To identify the effect of specific mutations within exons 14 and 26, two minigenes were employed, one carrying genomic sequences from exon 13 through exon 15 and the other carrying genomic sequences from exon 25 through exon 27. As expected, insertion of pathogenic missense mutations in the gRNA-targeted regions promoted skipping of *POLR3A* exons 14 and 26 (Figure 2). Interestingly, despite being separated by just three nucleotides, 1797G>C and 1800C>T mutations produced different degrees of skipping of exon 14. While 1797G>C caused moderate skipping of exon 14, 1800C>T triggered almost complete skipping. These results underscore the complexity of splicing regulation in which different positions within a given motif may have different impact on splicing. Similar to 1800C>T, 3392A>G mutation triggered massive skipping of exon 26. Effect of missense mutations on splicing of exons 14 and 26 was captured in all three cell lines employed, including EcR-293, human oligodendroglioma, and MO3.13 cells, supporting that splicing of these exons is not driven by cell-specific factors.

It is expected that the consequences of a mutation within an ESE or ISE could be reversed by a mutation or sequestration of an ESS or ISS. To identify potential ESSs/ISSs, the authors employed gRNAs targeting intronic sequences upstream and downstream of exons 14 and 26 of *POLR3A* minigenes carrying pathogenic missense mutations 1797G>C, 1800C>T, and 3392A>G. Results revealed multiple intronic regions targeting of which by gRNAs had varying degrees of stimulatory effect on splicing of exons 14 and 26. The strongest stimulatory effect was captured with I13-5 and I25-5 gRNAs that targeted intronic sequences ∼60 nt upstream of exons 14 and 26, respectively. There was near total restoration of exon 14 inclusion by I13-5 gRNA in the case of mild 1797G>C mutation (Figure 2). However, 1800C>T and 3392A>G being severe mutations, there was only partial restoration of inclusion of exons 14 and 26 by I13-5 and I25-5 gRNAs, respectively.

The next set of experiments evaluated the effect of ASOs targeting sequences corresponding to annealing positions of I13-5 and I25-5 gRNAs. The authors used 24-mer and 16-mer ASOs encompassing different modifications at the 2′-hydroxyl positions. While 24-mer incorporated 2′O-methyl (2′OMe) modifications, 16-mer ASOs incorporated alternate 2′OMe and 2′O-methoxyethyl (2′MOE) modifications. All ASOs targeting sequences corresponding to annealing positions of I13-5 and I25-5 gRNAs showed a stimulatory effect on splicing of the downstream exons carrying pathogenic mutations. Similar to the results observed with gRNAs, the stimulatory effect of ASOs in the case of severe 1800C>T and 3392A>G mutations were modest (Figure 2). Overall, these findings confirmed the proof of principle that the site-specific recruitment of gRNAs complexed with dCas13Rx could be exploited for initial uncovering of splicing regulatory elements as potential therapeutic targets for an ASO-mediated splicing correction. A potential drawback of the dCas13d/gRNA-based approach is the lack of granularity in defining the boundaries of regulatory elements due to long length of gRNAs for Cas13 variants, which may require targeting of each base of interest with multiple gRNAs or doing further validations with minigenes.^6^ Additional caveat is the ASO size and its position of annealing that could dramatically change the outcome of splicing.^9^^,^^10^ The authors acknowledge that the size and the chemical modification of a therapeutic ASO would have to be carefully tailored for specific mutations. Given the clinical heterogeneity and the large number of missense variants described in POLR3A-associated leukodystrophies and neurological disorders, systematic mapping of mutation-induced splicing defects in POLR3A represents a critical step toward precision RNA-targeted therapeutic development. Taken as a whole, findings presented by Shkreta et al. lay a strong foundation for a better understanding of splicing regulation and therapeutic intervention pertaining to the pathological conditions associated with a vast array of poorly understood missense mutations.

Beyond its role as a screening platform, RNA-targeting Cas13 systems may also hold potential as direct therapeutic agents for splicing modulation. Catalytically inactive Cas13 variants can be programmed to bind specific pre-mRNA regions and influence exon inclusion or skipping, offering a potentially durable alternative to antisense oligonucleotides through vector-mediated delivery. In principle, such approaches could enable sustained and tunable splicing correction. However, important challenges remain, including efficient delivery, control of expression levels, immunogenicity, and minimization of off-target RNA interactions. Although ASOs currently represent a more clinically established modality, continued refinement of RNA-targeting CRISPR technologies may expand the therapeutic toolkit for precision RNA modulation.

## Acknowledgments

R.N.S. was supported by grants from the 10.13039/100000002National Institutes of Health (NIH; USA) NS055925 and NS136717.

## Declaration of interests

The antisense target ISS-N1 (patent US7838657) was discovered in the Singh laboratory at UMass Medical School (MA, USA). Inventors, including R.N.S. and UMASS Medical School, are currently benefiting from licensing of the ISS-N1 target to Ionis Pharmaceuticals. C.R.R.A. is an inventor on patent applications describing genome engineering technologies to treat SMA and other diseases. C.R.R.A. was a consultant for Biogen and holds stocks in publicly traded companies developing gene therapies.
